# A Novel CXCR4 antagonist enhances angiogenesis *via* modifying the ischaemic tissue environment

**DOI:** 10.1111/jcmm.13150

**Published:** 2017-04-04

**Authors:** Xiaoqing Yan, Xiaozhen Dai, Luqing He, Xiao Ling, Minglong Shao, Chi Zhang, Yuehui Wang, Jian Xiao, Lu Cai, Xiaokun Li, Yi Tan

**Affiliations:** ^1^ Chinese‐American Research Institute for Diabetic Complications Wenzhou Medical University Chashan University‐town Wenzhou Zhejiang China; ^2^ Pediatric Research Institute Department of Pediatrics University of Louisville Louisville KY USA; ^3^ Chinese‐American Pediatric Research Institute at the First Hospital of Wenzhou Medical University Wenzhou Zhejiang China; ^4^ School of Biomedicine Chengdu Medical College Chengdu Sichuan China; ^5^ Department of Geriatric Medicine the first hospital of Jilin university Changchun Jilin China

**Keywords:** P2G, endothelial progenitor cells, angiogenesis, AMD3100

## Abstract

Endothelial progenitor cells (EPCs) play a capital role in angiogenesis *via* directly participating in neo‐vessel formation and secreting pro‐angiogenic factors. Stromal cell‐derived factor 1 (SDF‐1) and its receptor CXCR4 play a critical role in the retention and quiescence of EPCs within its niche in the bone marrow. Disturbing the interaction between SDF‐1 and CXCR4 is an effective strategy for EPC mobilization. We developed a novel CXCR4 antagonist P2G, a mutant protein of SDF‐1β with high antagonistic activity against CXCR4 and high potency in enhancing ischaemic angiogenesis and blood perfusion. However, its direct effects on ischaemic tissue remain largely unknown. In this study, P2G was found to possess a robust capability to promote EPC infiltration and incorporation in neo‐vessels, enhance the expression and function of pro‐angiogenic factors, such as SDF‐1, vascular endothelial growth factor and matrix metalloprotein‐9, and activate cell signals involved in angiogenesis, such as proliferating cell nuclear antigen, protein kinase B (Akt), extracellular regulated protein kinases and mammalian target of rapamycin, in ischaemic tissue. Moreover, P2G can attenuate fibrotic remodelling to facilitate the recovery of ischaemic tissue. The capability of P2G in direct augmenting ischaemic environment for angiogenesis suggests that it is a potential candidate for the therapy of ischaemia diseases.

## Introduction

Endothelial progenitor cells (EPCs) are a kind of vascular progenitor 1, 2 that are derived from bone marrow 3. They widely exist in human cord blood, peripheral blood, vessel wall and heart tissue 4. EPCs participate in angiogenesis and the repair of injured an endothelium. EPCs facilitate ischaemic angiogenesis by directly incorporating into ischaemic sites to form neo‐vessels 4, and/or in a paracrine fashion by secreting angiogenic factors 5. Decreases and/or dysfunction of EPCs in some pathologic conditions, such as diabetes 6, 7, attenuate angiogenesis and repair of the endothelium. Local or systemic transplantation of EPCs from bone marrow 8, cord blood 9 or peripheral blood 10 can enhance ischaemic neovascularization and improve the function of ischaemic tissues in animals with limb or myocardial ischaemia. Considering its critical role in angiogenesis, EPC mobilization and/or transplantation have been taken as important therapy strategies in ischaemic diseases.

Stromal cell‐derived factor‐1 (SDF‐1), also known as CXCL12, and its receptor, CXCR4, are implicated in the migration 11, chemotaxis, homing 12, 13 and survival 14, 15 of EPCs. The SDF‐1‐CXCR4 axis plays a principal role in the retention of EPCs within its niche in bone marrow 16, 17, which suggests that antagonizing interactions between SDF‐1 produced by bone marrow stromal cells and CXCR4 expressed in EPC, or perturbing the SDF‐1 gradient between bone marrow and peripheral circulation may effectively mobilize EPCs 18. Granulocyte colony‐stimulating factor (GC‐SF) is the ‘gold‐standard’ reagent in mobilizing hematopoietic stem cells/hematopoietic progenitor cells (HSCs/HPCs) 19. It can decrease SDF‐1 expression in the bone marrow to form a SDF‐1 gradient between bone marrow and peripheral circulation, which will induce EPC mobilization 20. However, this process took several days and the effect of GC‐SF has broad interindividual variability 21. AMD3100, a representative CXCR4 antagonist, can effectively and rapidly mobilize HSCs, including EPCs 22. However, HSCs mobilized by AMD3100 showed obviously impaired survival after transplantation 23, and severe cardiac toxicity has been reported after long‐term AMD3100 administration 24. Hence, the search for novel reagents for EPC mobilization and homing is still necessary.

We have developed a novel peptide antagonist against CXCR4, named P2G, by replacing the *N*‐terminal second proline residue of human SDF‐1β with glycine 25. Our previous study had demonstrated that P2G can potently antagonize CXCR4 and improve blood flow reperfusion, angiogenesis and muscle regeneration in a mouse hind limb ischaemic (HLI) model, while had no apparent adverse effects on the heart, liver, kidney and testis even after administration for 14 days 25. However, its direct effects on ischaemic tissue remain largely unknown. The aim of this study was to determine the direct effects of P2G on ischaemic tissue in a rat HLI model.

## Materials and methods

### Animals

Male Sprague‐Dawley (SD) rats (8 weeks old) were purchased from the Harlan (Harlan Laboratories, Inc., Indianapolis, IN, USA) and maintained under specific pathogen‐free conditions at the University of Louisville Animal Facility (Louisville, Kentucky, USA). All experiments were approved by the Animal Care and Use Committee of the University of Louisville.

### Experimental protocol

Dose effect of P2G on white blood cells (WBCs) mobilization was investigated, as described in a previous report 23. Rats were divided into eight groups. Different dosages of P2G ranged from 0.049 to 3.15 μmol/kg body weight were prepared in phosphate‐buffered saline (PBS) and injected intravenously. PBS was used as negative control. One hour after injection, rats were sacrificed and peripheral blood was collected with heparin conjugated tube (BD, Franklin Lakes, NJ, USA). Total white blood cells, lymphocytes, monocytes, neutrophils, eosinophils and basophils were counted using a hemocytometer (HEMAVET 950FS, DREW Scientific Inc., Oxford, CT, USA).

A HLI model was induced, as described in our previous study 25, for evaluating the effect of P2G on ischaemic angiogenesis. Briefly, rats were anesthetized with an intraperitoneal injection of ketamine and xylazine. Then, both the artery and vein were ligated and removed at two ends of the femoral artery and vein in the right hind limb. Twenty minutes after surgery, PBS, P2G or AMD3100 were administrated by intravenous injection, respectively. The optimal dose of P2G for WBCs mobilization was chosen to evaluate the effect of P2G on ischaemic angiogenesis, and AMD3100 at the same dose level as P2G was chosen as a positive control. Rats were sacrificed at 1, 2 or 3 weeks post‐injection, and the gastrocnemius muscle was collected for further assay.

### Immunohistochemistry

Gastrocnemius muscle tissues fixed in 10% buffered formalin were dehydrated in a graded alcohol series, cleared with xylene, embedded in paraffin and sectioned at 5 μm thickness for pathological and immunohistochemical staining. Paraffin‐embedded sections of muscle tissues were dewaxed and then incubated in 1× Target Retrieval Solution (Dako, Carpinteria, CA, USA), heated to and kept at 98°C using a microwave oven for 15 min. to retrieve antigens. After endogenous peroxidase inactivation with 3% hydrogen peroxide for 10 min. at room temperature, sections were blocked with 5% bovine serum albumin ‎(BSA) for 30 min. then incubated with primary antibodies against CD31 (BD Bioscience) and CD34 (BD Bioscience) overnight at 4°C. After being washed with phosphate‐buffered saline (PBS), sections were incubated with corresponding horseradish peroxidase‐conjugated secondary antibodies (1:300–400 dilutions with PBS) for 1 hr at room temperature. For the colour development of immunohistochemical staining, sections were treated with a peroxidase substrate DAB kit (Vector Laboratories, Inc., Burlingame, CA, USA) and counterstained with haematoxylin.

### Immunofluorescence

Cryostat sections (5 μm) from ischaemic gastrocnemius muscles dissected from rats at weeks 1, 2 and 3 after the surgical procedure were air‐dried at room temperature for 30 min., fixed in ice‐cold acetone for 5 min. and then air‐dried for another 30 min. The slides were washed with three changes in PBS for 5 min. each and incubated with 5% BSA in PBS for 20 min. to suppress non‐specific binding of IgG. Then, the primary antibody cocktails: goat anti‐rat CXCR4 (Santa Cruz Biotechnology, Santa Cruz, CA, USA) was mixed with mouse anti‐rat CD31 monoclonal antibody at 1:50 dilution in PBS with 3% BSA, respectively, and then incubated for 1 hr. After being washed with PBS three times for 5 min. each, the slides were incubated for 45 min. with fluorochrome‐conjugated secondary antibodies (1:400) in PBS with 3% BSA in a dark chamber, followed by three washes with PBS containing 0.1% Triton X‐100, and coverslips were mounted with 90% glycerol in PBS. The expression of different proteins was examined using a fluorescence microscope with appropriate filters.

### Western blot

The expression and/or phosphorylation of specific antigens were assayed by Western blot, as in our previous reports 25, 26. Tissue samples (gastrocnemius muscle from ischaemic hind limbs), obtained at weeks 1, 2 and 3 after surgery, were thawed and homogenized in 500 μL of RIPA lysis buffer containing protease inhibitors (Santa Cruz Biotechnology). Proteins were separated in 8%, 10% or 12% SDS‐PAGE and then blotted onto a nitrocellulose membrane (Hybond ECL, Amersham). After blockade with 5% non‐fat milk for 1 hr, membranes were incubated overnight at 4°C in antibodies against vascular endothelial growth factor (VEGF;1:500) from Millipore, protein kinase B (Akt;1:1000), p‐Akt (1:1000), extracellular signal‐regulated kinases (ERK) (1:5000), p‐ERK (1:2000), mammalian target of rapamycin (mTOR, 1:1000), p‐mTOR (1:1000) and proliferating cell nuclear antigen (PCNA, 1:2000) from Cell Signaling Technology, and connective tissue growth factor (CTGF) (1:1000), SDF‐1 (1:1000), CXCR4 (1:1000) and β‐actin (1:2000) from Santa Cruz Biotechnology, respectively. Specific bands were then detected with HRP‐conjugated secondary antibodies. After three washes with TBST, antigen‐antibody complexes were visualized with an enhanced chemiluminescence detection kit (Thermo Scientific, Waltham, MA, USA). The expression and/or phosphorylation of specific target genes were expressed as the ratio of quantification of optical density of the specific protein band to that of β‐actin or the total target gene.

### Gelatin zymography

The activity of matrix metallopeptidase 9 (MMP‐9) was evaluated by gelatin zymography. In brief, protein lysis was mixed with equal amounts of non‐reducing Laemmli buffer and subjected to 10% sodium dodecyl sulphate–polyacrylamide gel electrophoresis (SDS‐PAGE) containing 0.1% gelatin (Sigma‐Aldrich, St. Louis MO, USA). After three washes with 2.5% Triton X‐100 for 1 hr each, gels were incubated for 12–16 hrs at 37°C in proteolysis buffer (50 mmol/L Tris, 50 mmol/L CaCl2, 0.5 mol/L NaCl, pH = 7.8) and stained with 0.25% Coomassie Blue R‐250 (Sigma‐Aldrich) in methanol‐acetic acid‐water (45:10:45). After destaining with the solvent of Coomassie Blue R‐250, MMP‐9 activity was detected as a clear zone on a blue gel. Photographs were taken from the gels, and the band intensity was quantified by densitometry.

### Sirius‐red staining for collagen

Fibrosis at the wound site was evaluated by Sirius‐red staining for collagen deposition, as described in our previous study 27, 28. Briefly, 5‐μm tissue sections were used for Sirius‐red staining with 0.1% Sirius‐red F3BA and 0.25% Fast Green FCF. Sections stained for Sirius‐red then were assessed for the proportion of collagen using a Nikon Eclipse E600 microscopy system.

### Statistical analysis

Data were initially analysed by one‐way anova, followed by a multiple difference analysis (*t*‐test). All data are presented as the mean ± S.D. *P *<* *0.05 was considered as a significant difference.

## Results

### P2G mobilizes WBCs in a dose‐dependent manner

We evaluated the dose–response effect of P2G on mobilization of WBCs of intact rats. Hemocytometer results showed that P2G mobilized WBCs (Fig. S1A), neutrophils (Fig. S1B), basophils (Fig. S1D) within the dose range from 0.197 to 3.15 μmol/kg body weight, and eosinophils (Fig. S1C) from 0.394 to 3.15 μmol/kg body weight. But P2G administration had no significant effects on monocyte number (Fig. S1E) and did not mobilize lymphocytes (Fig. S1F) except at the highest dose level tested in this study. These results demonstrated that P2G could dose‐dependently mobilize bone marrow cells in rats, and the optimal dose is 0.788 μmol/kg body weight. This dose was chosen to evaluate the effects of P2G on ischaemic angiogenesis in a rat HLI model in the following study.

### P2G promotes angiogenesis in HLI

**Figure 1:**
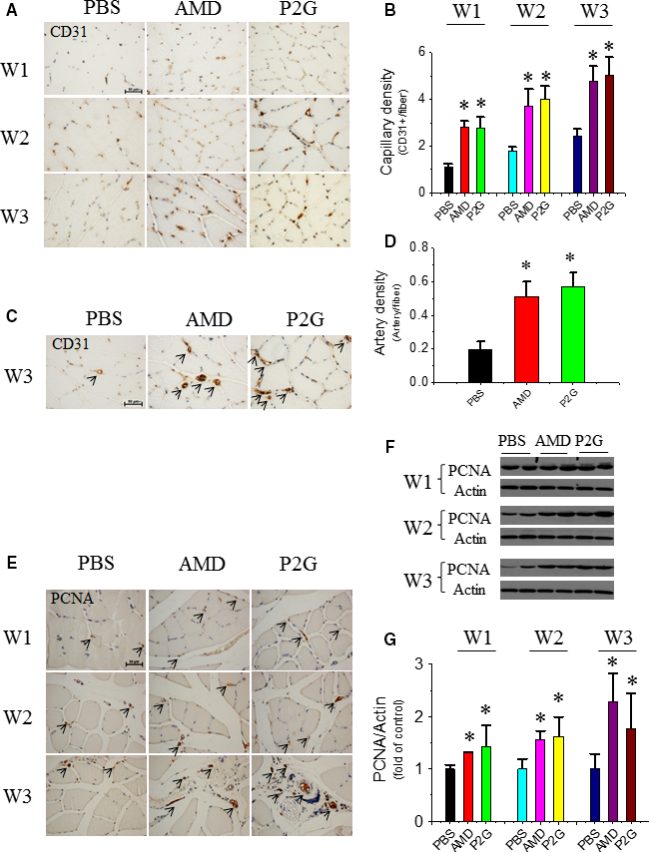
P2G enhances neo‐vessel formation in ischaemic gastrocnemius muscles. Representative microscopic photographs of capillaries (brown dots) identified by CD31 staining were provided (**A**), and the quantitative summaries of capillary density were expressed as capillary number per muscle fibre at the 1st (W1), 2nd (W2) and 3rd (W3) week after surgery (**B**); representative microscopic photographs of small blood vessels identified by CD31 staining were provided (**C**), and the quantitative summaries of small blood vessel density were expressed as vessel number per muscle fibre at W3 (**D**). Cell proliferation indicator PCNA was evaluated by immunohistochemical staining (**E**) and further confirmed by Western blot (**F**,** G**). *n *=* *5 for PBS, 4 for AMD and 8 for P2G group, respectively. *P <* *0.05 *versus* PBS, Bar = 50 μm. PBS: Phosphate‐buffered saline; AMD: AMD3100.

Capillary density in HLI rats was evaluated by immunohistochemical staining of CD31, an endothelial cell surface molecular marker. The results showed more than a threefold increase in capillary density in the muscles of AMD3100 (AMD) or P2G‐treated rats at 1, 2 and 3 weeks after treatment when compared with controls, and no significant difference in capillary density between AMD‐ and P2G‐treated groups was observed (Fig. 1A and B). Moreover, we found both the AMD and P2G treatment significantly increased the quantity of small blood vessels, which were indicated by circular CD31‐positive staining (Fig. 1C), at 3 weeks post‐treatment and no significant difference between AMD and P2G groups was observed (Fig. 1D). In addition, PCNA staining results indicated that both the AMD‐ and P2G‐treated group exhibited enhanced cell proliferation in capillary and small blood vessels in the gastrocnemius muscle tissue (Fig. 1E), which was further confirmed by a Western bot assay of PCNA expression (Fig. 1F and G). These results demonstrated that AMD and P2G treatment obviously stimulated new blood vessel formation.

### P2G increases EPC localization in the ischaemic gastrocnemius muscle

**Figure 2:**
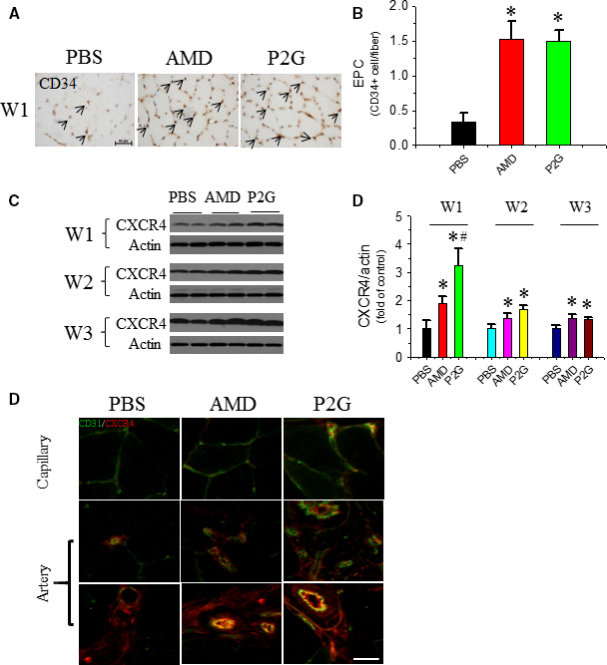
P2G enhances endothelial progenitor cell (EPC) infiltrating in ischaemic gastrocnemius muscles. At the indicated time‐points after surgery, EPC localization in the ischaemic gastrocnemius muscle tissue was identified by a EPC marker CD34 by immunohistochemical staining (**A**), and the quantitative analysis of EPC density was expressed as capillary number per muscle fibre at W1 (**B**); The expression of another EPC marker CXCR4 (**C**) in the ischaemic gastrocnemius muscle tissue was detected by Western blot assay, followed by quantitative analyses of the optical density relative to the loading control actin (**D**). The localization of CXCR4 +  EPC in capillary and small blood vessels was determined by co‐immunofluorescent staining of both CD31 and CXCR4 (**E**). *n *=* *5 for PBS, 4 for AMD and 8 for P2G group respectively. *P <* *0.05 *versus* PBS, ^#^ *P *<* *0.05 *versus* AMD3100, Bar = 50 μm.

To validate that improved new vessel formation was caused by enhanced EPC infiltration and incorporation, EPC localization in the ischaemic gastrocnemius muscle was detected by CD34 staining at 1 week after AMD or P2G administration. Immunohistochemistry showed that the CD34‐positive EPC number in the gastrocnemius muscle was elevated in both AMD3100‐ and P2G‐treated groups (Fig. 2A and B), and it was further confirmed by another EPC marker, the enhanced CXCR4 expression in ischaemic muscle tissue (Fig. 2C and D). Moreover, the co‐immunofluorescent staining (Fig. 2E) of CD31 (green) and CXCR4 (red) showed that CXCR4 staining partially overlapped with that of CD31, which revealed that CXCR4‐positive EPCs were partially incorporated in capillary and small blood vessels. Also, stronger CXCR4 staining in both AMD3100‐ and P2G‐treated groups indicated that more EPCs incorporated in the new blood vessels of AMD3100‐ and P2G‐treated rats than that of the PBS control group.

### P2G enhances SDF‐1 and VEGF expressions and MMP‐9 activity in the ischaemic gastrocnemius muscle

**Figure 3:**
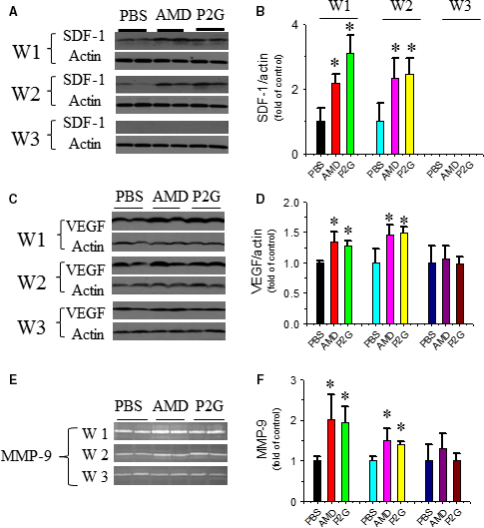
P2G up‐regulates pro‐angiogenic factors in ischaemic gastrocnemius muscles. The expression of SDF‐1 (**A**,** B**) and VEGF (**C**,** D**) was detected by Western blot, and the activity of MMP‐9 was detected by gelatin zymography (**E**,** F**). *n *=* *5 for PBS, 4 for AMD and 8 for P2G group, respectively. *P <* *0.05 *versus* PBS.

Pro‐angiogenic factors expressed in ischaemic tissue also contribute to angiogenesis. Western blot results showed that both P2G and AMD3100 increased SDF‐1 expression in the gastrocnemius muscle at 1 week and 2 weeks after administration, and its expression regressed to a very low basal level that was undetectable by Western blot at 3 weeks (Fig. 3A and B). Another pro‐angiogenic factor, VEGF, was also increased in the ischaemic tissue of both P2G and AMD3100‐treated groups at 1 week and 2 weeks after administration and then regressed to the basal level at 3 weeks (Fig. 3C and D). These results indicated that P2G may promote neovascularization by enhancing pro‐angiogenic factors expression in ischaemic tissues. Matrix remodelling is another important step in neo‐vessel formation, and MMP‐9 plays a critical role in this process. Gelatin zymography results showed that MMP‐9 activity in the ischaemic gastrocnemius muscle of both the P2G‐ and AMD3100‐treated groups was obviously higher than that of the PBS‐treated group at 1 week and 2 weeks after treatment (Fig. 3E and F), which revealed that P2G could also facilitate angiogenesis by promoting matrix remodelling in ischaemic tissue.

### P2G enhances angiogenic signal activation in ischaemic gastrocnemius muscle

**Figure 4:**
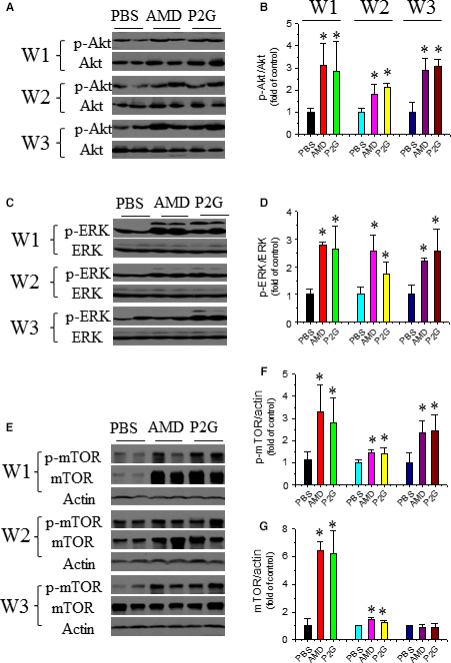
P2G enhances pro‐angiogenic signal activation in ischaemic gastrocnemius muscles. The expression and phosphorylation of Akt (**A**,** B**), ERK (**C**,** D**) and mTOR (**E**,** F**,** G**) were assayed by Western blot and quantified by optical density relative to the loading control actin and/or the total target protein, respectively. *n *=* *5 for PBS, 4 for AMD and 8 for P2G group, respectively. *P <* *0.05 *versus* PBS.

Besides pro‐angiogenic factors, the activation of several signal pathways involved in angiogenesis, including Akt, Erk1/2 and mTOR were also evaluated. Western blot results showed that the phosphorylation of Akt was up‐regulated by P2G or AMD at 1, 2 and 3 weeks after administration, and no obvious difference between the P2G group and AMD group was observed (Fig. 4A and B). The activation of Erk1/2 (Fig. 4C and D) and mTOR (Fig. 4E–G) showed a similar pattern. These results indicated that P2G administration might promote angiogenesis *via* activating angiogenic signal pathways in ischaemic tissue.

### P2G represses fibrotic remodelling in ischaemic gastrocnemius muscle

**Figure 5:**
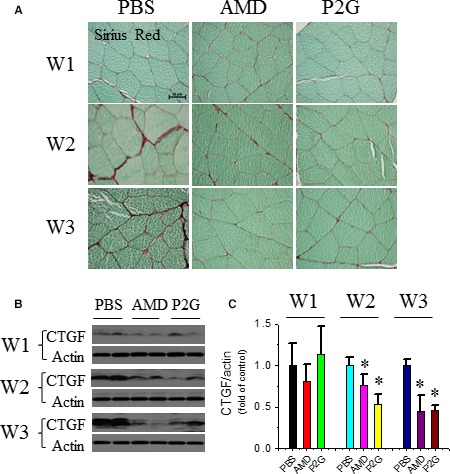
P2G attenuates fibrotic remodelling in ischaemic gastrocnemius muscles. The accumulation of collagen in ischaemic gastrocnemius muscles was assayed by Sirius‐red staining (**A**). The expression of fibrotic marker connective tissue growth factor (CTGF) in ischaemic gastrocnemius muscle was assayed by Western blot (**B**) and quantified by optical density relative to the loading control actin (**C**). *n *=* *5 for PBS, 4 for AMD and 8 for P2G group, respectively. *P <* *0.05 *versus* PBS.

Fibrosis is one of the major causes of muscle dysfunction, and ischaemic muscles suffer serious fibrosis accompanied by recovery. Sirius‐red staining showed that the PBS group had an aggravating collagen deposition in the ischaemic gastrocnemius muscle as time passed, which was ameliorated in the P2G or AMD3100‐treated groups. Collagen deposition in the ischaemic gastrocnemius muscle treated with P2G or AMD3100 was attenuated at 2 and 3 weeks after administration when compared with that of PBS (Fig. 5A). Connective tissue growth factor (CTGF) is a well‐documented indicator of fibrosis, and Western blot results showed that its expression in the P2G‐ or AMD3100‐treated groups was significantly lower than that of PBS at 2 and 3 weeks after ischaemia (Fig. 5B and C), which further proved that P2G can alleviate fibrosis in ischaemic tissue.

## Discussion

EPCs are a type of vascular progenitors that are curative for many life‐threatening disorders, especially for ischaemic diseases. Abundant animal experiments, pre‐clinic or clinical trials have proved that EPCs have a therapeutic effect to limb ischaemia 9, myocardial infarction 10 and stroke 29. Under normal conditions, EPCs maintains in a quiescent state in a bone marrow osteoblastic niche, which was largely attributed to the interaction between SDF‐1 produced by stromal cells and CXCR4 expresses in EPCs 17, 30, 31. Antagonizing the SDF‐1/CXCR4 axis or changing the SDF‐1 gradient between bone marrow and peripheral circulation was considered as an effective EPC mobilizing strategy 18. In addition to mobilizing EPC from its niche, promoting EPC function 32, 33 and trafficking EPCs in ischaemic tissue is also critical in angiogenesis. SDF‐1 expression induced by hypoxia‐inducible factor 1α (HIF‐1α) plays a principle role in trafficking EPCs in ischaemic tissue 34. HIF‐1α dysfunction under some pathologic conditions, such as diabetes, attenuates SDF‐1 up‐regulation and then impairs ischaemic angiogenesis 35, 36, while enhancing HIF‐1α expression 37, and/or its stabilization 38 can improve diabetic angiogenesis. These facts revealed that the modifying ischaemic tissue environment is another important strategy in promoting angiogenesis, especially under some chronic disease conditions, such as diabetes 39.

In a previous study, we developed a novel peptide antagonist against CXCR4, that is P2G, by replacing the *N*‐terminal second proline residue of human SDF‐1β with glycine, and demonstrated that P2G is an effective CXCR4 antagonist with the ability to improve ischaemic angiogenesis and muscle regeneration in a HLI mouse model 25. In the present study, its role in modifying an ischaemic tissue condition was investigated. First, the role of P2G in promoting ischaemic angiogenesis was determined. P2G administration to HLI rats can significantly increase the capillary (Fig. 1A and B) and artery (Fig. 1C and D) density in the ischaemic gastrocnemius muscle, which is comparable to that of AMD3100. These results were consistent with that in HLI mouse model 25 and further confirmed the potency of P2G in ischaemic diseases therapy. Meanwhile, P2G may also enhance EPC trafficking to facilitate ischaemic angiogenesis because P2G treatment can increase CD34^+^ (Fig. 2A and B) and CXCR4^+^  (Fig. 2C–E) EPC retention in the ischaemic gastrocnemius muscle, which is even more efficient than that of AMD3100.

EPC trafficking in ischaemic tissue is majorly regulated by SDF‐1 34, the ligand of CXCR4. P2G treatment can significantly enhance SDF‐1 expression in ischaemic gastrocnemius muscle, which was even more efficient than that of AMD3100 at the 1st week after ischaemia (Fig. 3A and B). Other than SDF‐1, some other pro‐angiogenic factors, such as VEGF (Fig. 3C and D) and MMP‐9 (Fig. 3E and F), were also up‐regulated in the ischaemic gastrocnemius muscle after P2G administration. VEGF can enhance EPC homing and promote nearby endothelial cell (EC) migration and proliferation 40, while MMP‐9 facilitates neo‐vessel formation by promoting matrix remodelling 41. Up‐regulation of VEGF and MMP‐9 can both contribute to P2G facilitating EPC retention and angiogenesis.

In addition to these growth factors, some cell signals involved in angiogenesis are also activated in gastrocnemius muscles after P2G treatment. PCNA is a typical marker of cell proliferation. P2G can enhance PCNA expression in the ischaemic gastrocnemius muscle (Fig. 1E–G). Moreover, the activation of Akt (Fig. 4A and B), ERK (Fig. 4C and D) and mTOR (Fig. 4E–G), several key signals involved in cell survival, are also enhanced by P2G, which are comparable with that of AMD3100 treatment. Although the underlying mechanism needs to be further clarified, the activation of these pro‐angiogenic signals also demonstrated the capability of P2G in modifying the ischaemic tissue environment.

Fibrosis is another challenge for recovery of ischaemia tissues. Decreases in collagen deposition (Fig. 5A) and CTGF expression (Fig. 5B and C) revealed that P2G can alleviate fibrosis in the ischaemic gastrocnemius muscle, which further confirmed the therapeutic capability of P2G in modifying the ischaemic tissue environment and promoting ischaemic tissue recovery.

**Figure 6:**
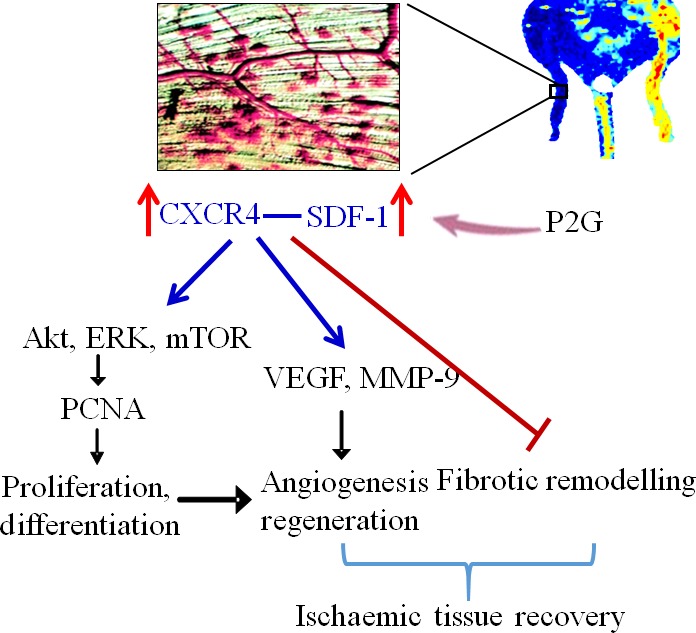
Schematic diagram of P2G promoting ischaemic angiogenesis. P2G treatment enhances SDF‐1 production and CXCR4 expression in the ischaemic tissue. The interaction of SDF‐1 and CXCR4 up‐regulates VEGF expression and MMP‐9 activity, which promotes angiogenesis and tissue regeneration. Moreover, the interaction between SDF‐1 and CXCR4 also activates Akt, ERK and mTOR pathway, then up‐regulates PCNA expression and further enhances cell proliferation and differentiation, which contribute to angiogenesis and tissue regeneration, resulting in ordered remodelling. [Colour figure can be viewed at wileyonlinelibrary.com]

In conclusion, P2G treatment can promote angiogenesis in a HLI model, which may partially be attributed to its ability to modify the ischaemic tissue environment, including up‐regulation of pro‐angiogenic factors, such as SDF‐1, VEGF, MMP‐9, and activating the signal pathways involved in angiogenesis, such as PCNA, Akt, ERK and mTOR in ischaemic tissue (Fig. 6). Moreover, P2G can attenuate fibrotic remodelling in ischaemic tissue. These results indicate that P2G is a potential candidate for the therapy of ischaemia diseases.

## Conflict of interest

None.

## Supporting information


**Fig. S1** The dose effect of P2G on white blood cells mobilization in intact rats.
