# Supplementary material for: A Novel CXCR4 antagonist enhances angiogenesis via modifying the ischaemic tissue environment
Source: J Cell Mol Med. 2017 Apr 4;21(10):2298–307. doi: 10.1111/jcmm.13150 (PMC5618675; doi:10.1111/jcmm.13150)
Supplement: Supplementary file 1 — Fig. S1 The dose effect of P2G on white blood cells mobilization in intact rats. [file JCMM-21-2298-s001.pptx]

## Slide 1
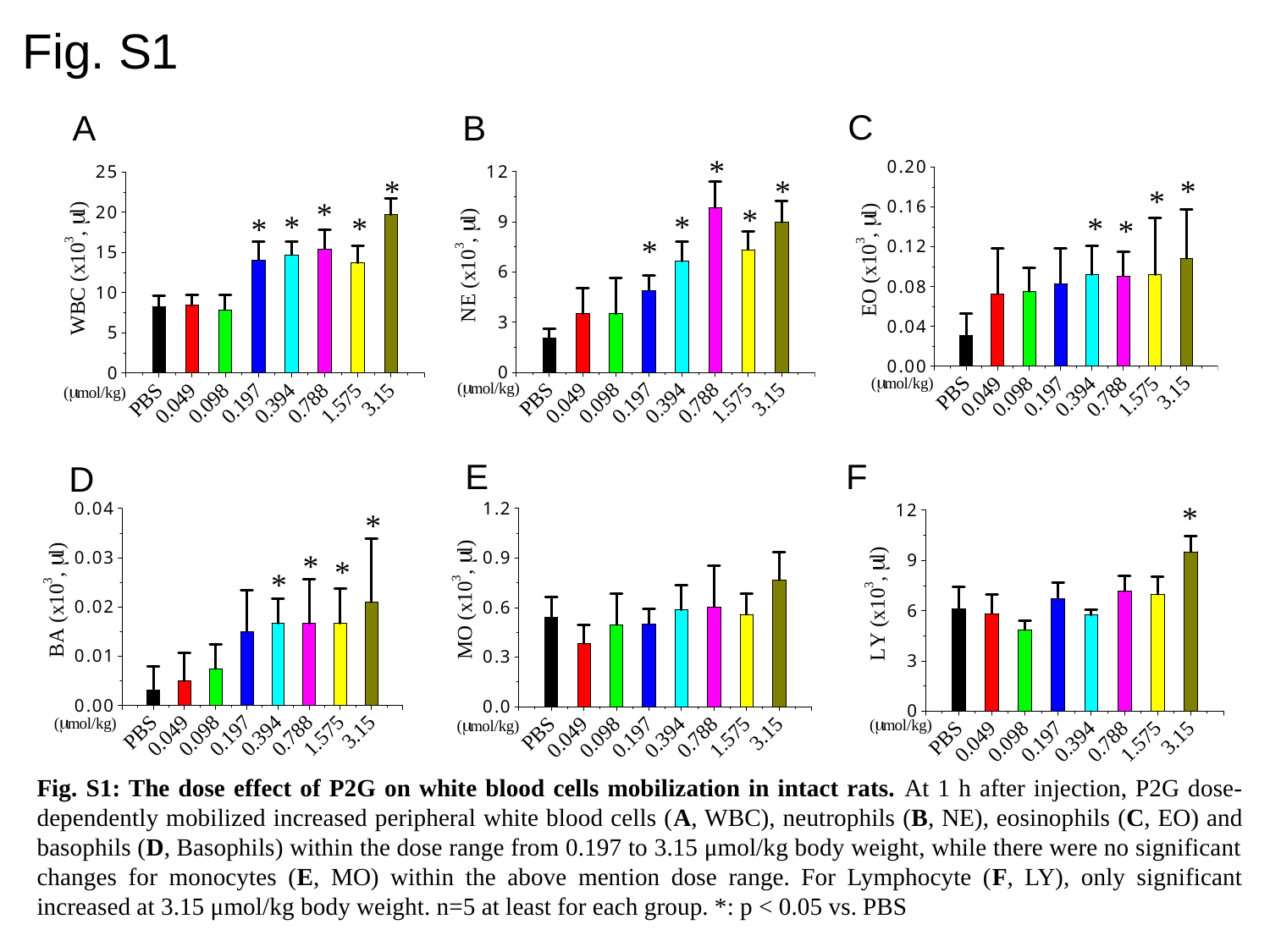

Fig. S1
C
A
B
E
F
D
Fig. S1: The dose effect of P2G on white blood cells mobilization in intact rats. At 1 h after injection, P2G dose-dependently mobilized increased peripheral white blood cells (A, WBC), neutrophils (B, NE), eosinophils (C, EO) and basophils (D, Basophils) within the dose range from 0.197 to 3.15 μmol/kg body weight, while there were no significant changes for monocytes (E, MO) within the above mention dose range. For Lymphocyte (F, LY), only significant increased at 3.15 μmol/kg body weight. n=5 at least for each group. *: p < 0.05 vs. PBS
